# Isolation Driven Divergence in Osmoregulation in *Galaxias maculatus* (Jenyns, 1848) (Actinopterygii: Osmeriformes)

**DOI:** 10.1371/journal.pone.0154766

**Published:** 2016-05-11

**Authors:** Ignacio Ruiz-Jarabo, Claudio A. González-Wevar, Ricardo Oyarzún, Juan Fuentes, Elie Poulin, Carlos Bertrán, Luis Vargas-Chacoff

**Affiliations:** 1 Centre of Marine Sciences (CCMar), Universidade do Algarve, Campus de Gambelas, 8005–139 Faro, Portugal; 2 GAIA Antártica, Universidad de Magallanes, Avenida Bulnes 01855, Punta Arenas, XII Región de Magallanes y la Antártica Chilena, Chile; 3 Laboratorio de Ecología Molecular, Instituto Milenio de Ecología y Biodiversidad (IEB), Departamento de Ciencias Ecológicas, Facultad de Ciencias, Universidad de Chile, Las Palmeras #3425, Ñuñoa, Santiago, Chile; 4 Instituto de Ciencias Marinas y Limnológicas, Facultad de Ciencias, Universidad Austral de Chile, casilla 567, Valdivia, Chile; 5 Centro Fondap de Investigación de Altas Latitudes (IDEAL), Universidad Austral de Chile, casilla 567, Valdivia, Chile; James Cook University, AUSTRALIA

## Abstract

**Background:**

Marine species have colonized extreme environments during evolution such as freshwater habitats. The amphidromous teleost fish, *Galaxias maculatus* is found mainly migrating between estuaries and rivers, but some landlocked populations have been described in lakes formed during the last deglaciation process in the Andes. In the present study we use mtDNA sequences to reconstruct the historical scenario of colonization of such a lake and evaluated the osmoregulatory shift associated to changes in habitat and life cycle between amphidromous and landlocked populations.

**Results:**

Standard diversity indices including the average number of nucleotide differences (*Π*) and the haplotype diversity index (*H*) indicated that both populations were, as expected, genetically distinctive, being the landlocked population less diverse than the diadromous one. Similarly, pairwise G_ST_ and N_ST_ comparison detected statistically significant differences between both populations, while genealogy of haplotypes evidenced a recent founder effect from the diadromous stock, followed by an expansion process in the lake. To test for physiological differences, individuals of both populations were challenged with a range of salinities from 0 to 30 ppt for 8 days following a period of progressive acclimation. The results showed that the landlocked population had a surprisingly wider tolerance to salinity, as landlocked fish survival was 100% from 0 to 20 ppt, whereas diadromous fish survival was 100% only from 10 to 15 ppt. The activity of ATPase enzymes, including Na^+^/K^+^-ATPase (NKA), and H^+^-ATPase (HA) was measured in gills and intestine. Activity differences were detected between the populations at the lowest salinities, including differences in ATPases other than NKA and HA. Population differences in mortality are not reflected in enzyme activity differences, suggesting divergence in other processes.

**Conclusions:**

These results clearly demonstrate the striking adaptive changes of *G*. *maculatus* osmoregulatory system, especially at hyposmotic environments, associated to a drastic shift in habitat and life cycle at a scale of a few thousand years.

## Introduction

Late Pleistocene processes in southern South America formed natural dams by melted ice masses after the Last Glacial Maximum (LGM), *c*. 18 ky BP (kiloyear before present) [1]. The outflow of these melted ice masses from the Patagonia to the Pacific Ocean across the Andes, about 13.2 ky BP, occurred when climatic warming along with tectonic and volcanic events modified the drainage systems. In the Chilean Central Andes it has been described the formation of isolated high mountain lakes associated to the last deglaciation in that period [2, 3]. During those processes, some diadromous species became landlocked to these lakes with little or no outlet to the sea [4].

The emergence of new ecological niches is often preceded by the attempt of some species to colonize them. One of the most drastic transitions for life is carried out while trying to breach the boundaries of hyposmotic habitats [5]. Thus, freshwater conditions require deep modifications at several levels. Many changes have been studied in marine to freshwater evolutionary transitions in fish, like displacements in reproductive age [6], endocrine readjustments [7]or even behavioral responses [8].Furthermore, the uptake of ions is of vital importance in freshwater [9] due to the osmotic differences between internal body fluids and external media. Hyper-osmoregulatory mechanisms in such hostile environments forced the individuals to counteract the passive loss of ions and gain water [10]. Although the colonization of FW habitats by SW fish has been reviewed recently [11], there is still a gap of information available about the osmoregulatory drift of a teleost fish when forced to modify its environmental salinity conditions for a few thousand generations.

In this sense, aquatic animals have developed a series of osmoregulatory strategies that allowed them to acclimate to different environmental salinities. These homeostatic actions are well described in fish [12], being gills and intestine the most important osmoregulatory tissues [13]. It should be noted that the Na^+^/K^+^-ATPase (NKA) holds the driving force for ion uptake from (or pumping out to) the environment in those epithelia [14]. Moreover, as this enzyme is located in the basolateral side of epithelial cells in the gills [15] and intestinal enterocytes [16], its major roles are related to the transport of ions in collaboration with other ATPases and ion carriers placed in the apical side. In any event, the capture of ions from the external media in freshwater habitats has historically been assigned to the vacuolar-type H^+^-ATPase (HA), which function seems to be critical for ion uptake from dilute media in many taxa [5, 17]. This enzyme generates a H^+^ gradient across the apical membrane, promoting the transport of other cations into the cell via other transporters. In this sense, previous studies analyzed the importance of those two pumps in fish acclimated to a range of environmental salinities [16, 18], being finally assumed that their relative importance varies greatly not only with the species, but also with their life stage [13].

*Galaxias maculatus* has been widely studied in terms of evolution [19, 20]as it is present in New Zealand, Australia, Tasmania, Chatman Island and South America [21], being considered one of the naturally greatest geographic distributions for a small diadromous fish in the planet [22]. The species, although considered as a freshwater one, is actually a diadromous organism because it breeds in estuaries, living the pre-metamorphic larva for up to 6 months in the sea [23]. However, some landlocked populations have been also described which complete life cycles occur on freshwater ecosystems [4]. Though considering the wide range of osmotic conditions this species have to afford during its life cycle, only a few osmoregulatory approaches have been performed [24]. *G*. *maculatus* can also tolerate prolonged drought periods, high temperatures, low pH values [25], a wide range of environmental salinities [26] and even periods of emersion [27]. These characteristics make this species an interesting model for research.

In this study, we examined the activity of the most important ionic pumps in gills and intestine (NKA, HA and Ouabain/Bafilomycin-insensitive ATPases) in the context of evolutionary change during freshwater adaptation of a diadromous population of *G*. *maculatus*. Our goal is to determine if these enzymes could evolve differently when submitting fish from originally freshwater-landlocked and diadromous populations to a wide range of environmental salinities. Previously, by the use of mitochondrial D-loop markers analyses, we compare the genetic diversity of a freshwater-landlocked and a diadromous populations and performed population demographic inferences in order to evaluate the historical process of colonization of a lake formed after the LGM.

## Results

The whole D-loop data set in *G*. *maculatus* included 55 individuals and consisted in 925 nucleotide positions. Considering that the D-loop is non-coding and highly variable mitochondrial region, several insertion and deletions were detected that were not considered for further analyses. Sequences were A-T rich (58.2%) compared to G-C content (41.8%). The diadromic population of *G*. *maculatus* (Valdivia River) showed high levels of genetic diversity with 70 polymorphic sites. Most of them (n = 62) were parsimoniously informative and D-loop sequences in this population were not saturated (Table 1). In contrast, the landlocked population (Colico Lake) showed lower levels of genetic diversity, only 18 positions were variable and 3 of them were parsimoniously informative. Again, D-loop sequences of the landlocked were not saturated at any position. Levels of genetic diversity measured through standard indices were higher in the diadromous population than in the landlocked one. For instance, haplotype diversity was higher in Valdivia River (*H =* 0.989) than in Colico lake (*H* = 0.840). Similarly, the average number of nucleotide differences (*Π*) and nucleotide diversity (*π*) were 25.04/0.0273 in Valdivia River and 1.79/0.0018 in Colico lake. Both, N_ST_ and G_ST_ comparisons between migratory and resident populations of *G*. *maculatus* showed significant differences (P = 0.000).

**Table 1:** Diversity indices, neutrality tests and mismatch distributions in landlocked and migratory populations of *Galaxias maculatus*.

Locality	*n*	*k*	*H*	*S*	*Π*	*π*	Tajima´s D	Fu´s FS	M.D
Colico Lake	27	16	0.840	18	1.79	0.0018	-2.29**	-14.48***	U
Valdivia River	28	24	0.989	70	25.04	0.0273	0.89	-3.70*	M
**Total**	**55**	**40**	**0.960**	**79**	**24.42**	**0.026**	**0.79**	-6.76***	**M**

Where: n = analyzed specimens; k = haplotype number; S = polymorphic sites; H = haplotype diversity; *Π =* average number of nucleotide differences; *π =* nucleotide diversity. M.D. = Mismatch distribution, U = Unimodal; M = Multimodal.

*p<0.05

**p<0.01

*** p<0.001.

**Fig 1:**
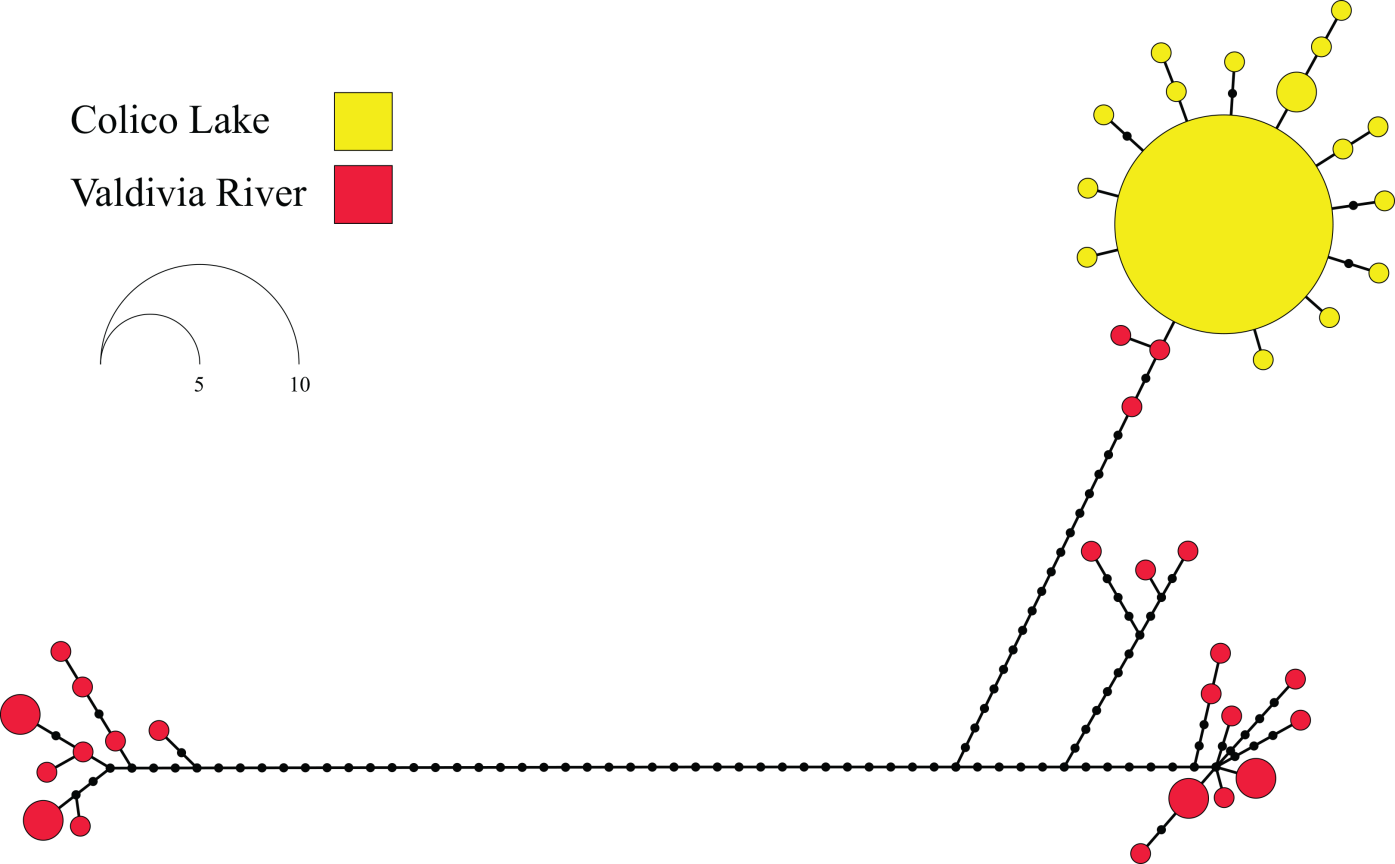
Maximum Parsimony Haplotype Network: including 55 individuals of Galaxias maculatus collected from a migratory population (Valdivia River) and a resident one (Colico Lake). Each mtDNA D-loop haplotype is represented by a colored circle indicating the locality where it was collected (Red = Valdivia River; Yellow = Colico Lake). The size of the circles is proportional to its frequencies in the whole data set. Small black circles indicate mutational steps along the genealogy.

**Fig 2:**
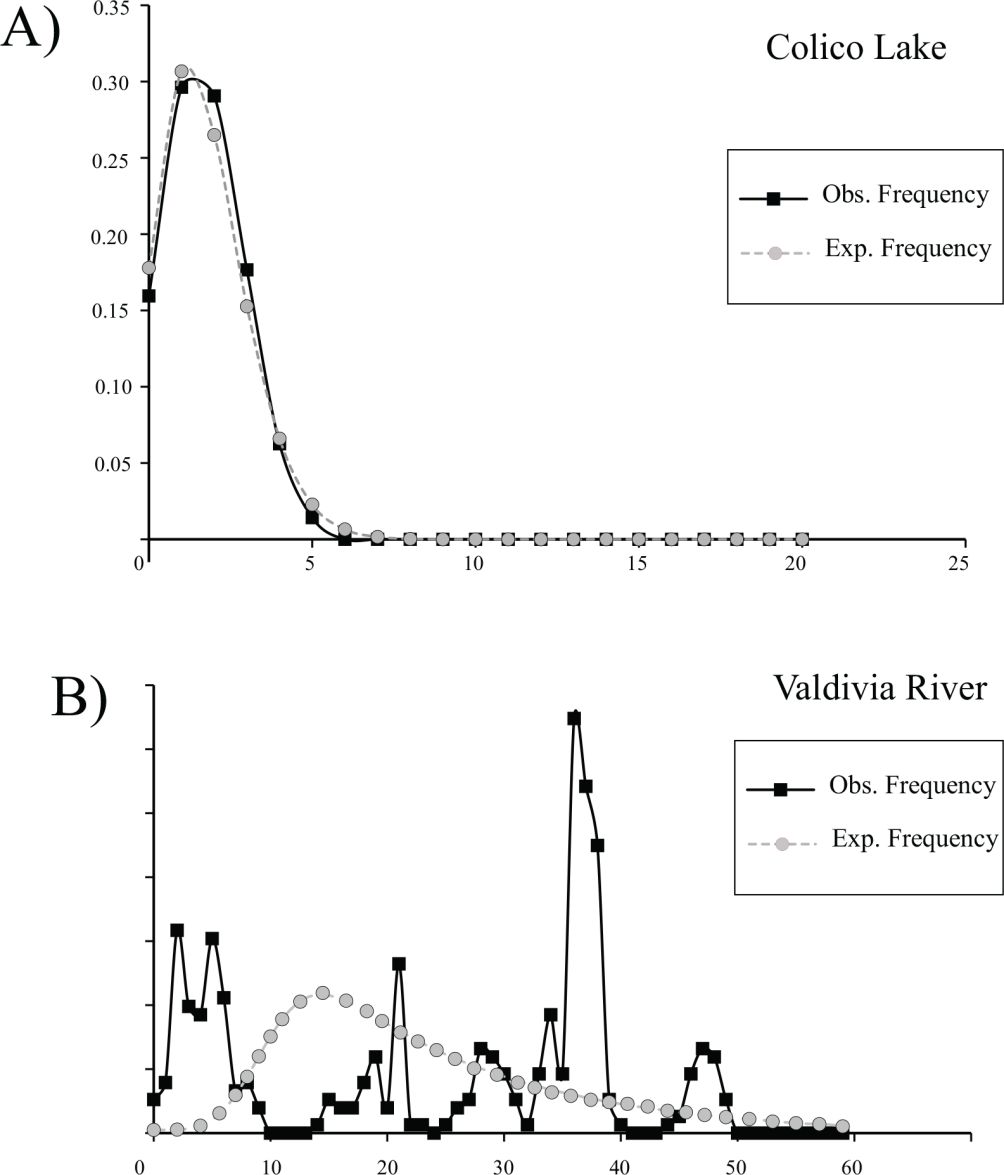
**Distribution of pairwise differences**: in *G*. *maculatus* populations from A) Colico Lake and B) Valdivia River.

**Fig 3:**
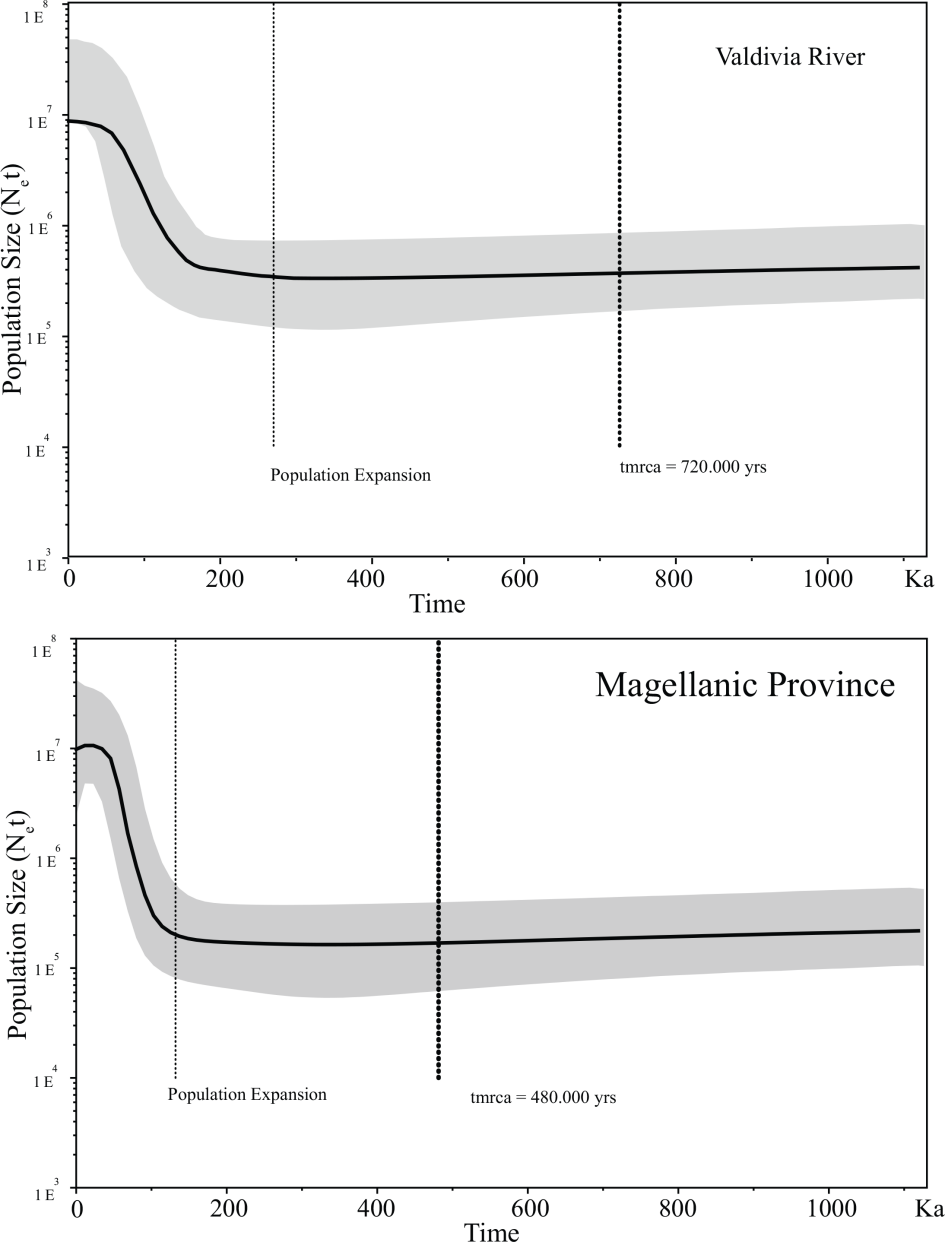
**Bayesian Skyline plot analyses revealing the times of the most recent common ancestor (tmrca):** for the Valdivia Rive (A) and Colico Lake (B) populations.

Maximum Parsimony haplotype network in *G*. *maculatus* recorded a total of 40 different haplotypes with an expanded genealogy (Fig 1). A total of 34 haplotypes (85%) were unique and only 6 haplotypes were present shared by two or more than two individuals. As previously recognized through mean standard diversity indices, Valdivia population showed a very expanded genealogy compared to the one registered in the Colico lake. In fact, Colico was characterized by a star-like topology with a dominant haplotype present in 41% of the individuals. As stated before [28], this haplotype should correspond to the most ancestral one in the Colico Lake, whereas the most derived ones are linked to it with a maximum branch length of three mutational steps. As expected, considering the contrasting patterns in terms of genetic diversity and genealogies recorded in both localities, Tajima’s D and Fu’s F_S_ neutrality tests showed dissimilar results between Valdivia and Colico. Tajima’s D test was negative and significant at Colico Lake and positive and non-significant at Valdivia (Table 1). In contrast, more sensitive Fu’s F_S_ test was negative and statistically significant for both, Valdivia and Colico. Similarly, analyses of pairwise differences in *G*. *maculatus* recovered a multimodal distribution in Valdivia while Colico showed a unimodal one (Fig 2A and 2B). Bayesian Skyline plot analyses recognized differences in the times of the most recent common ancestor (tmrca) and population expansions between Valdivia and Colico. Based on these analyses, the tmrca of Valdivia occurred ∼180 ky (Fig 3A) while the tmrca for Colico occurred ∼16 ky (Fig 3B). Similarly, the onset of the population expansion in Valdivia occurred ∼ 80,000years against the ∼ 7,000years estimated for the Colico Lake one.

Biometric parameters such as length, weight, condition factor (K) and residuals condition index of the individuals are shown in Table 2. The individuals from both populations were part of the same cohort, as they were early post-metamorphic juveniles with a minimum skin pigmentation and 30 to 60.8 mm length [29]. There were also differences (p<0.05) in the somatic growth parameters analyzed between the two populations studied here, as was described before for two osmotically distinctive populations of *G*. *maculatus* [30]. Thereupon, these circumstances allow us to compare the results obtained between the two populations, as it was previously described that the life stage could act as a factor of variance [13].

**Table 2:** Length, weight, Fulton´s condition factor (K) and residuals condition index (RCI):of two different populations (estuary and lake) of juvenile *G*. *maculatus* submitted to different experimental salinities. Values are mean ± SEM (N = 31–36).

	Estuary	Lake
**Length (cm)**	4.2 ± 0.1 *	5.7 ± 0.2
**Weight (g)**	0.36 ± 0.02 *	0.93 ± 0.13
**K (%)**	0.47 ± 0.01 *	0.42 ± 0.01
**RCI**	0.04 ± 0.02 *	-0.04 ± 0.03

^*^Statistical differences between populations (Student´s *t*-test, p<0.05).

**Fig 4:**
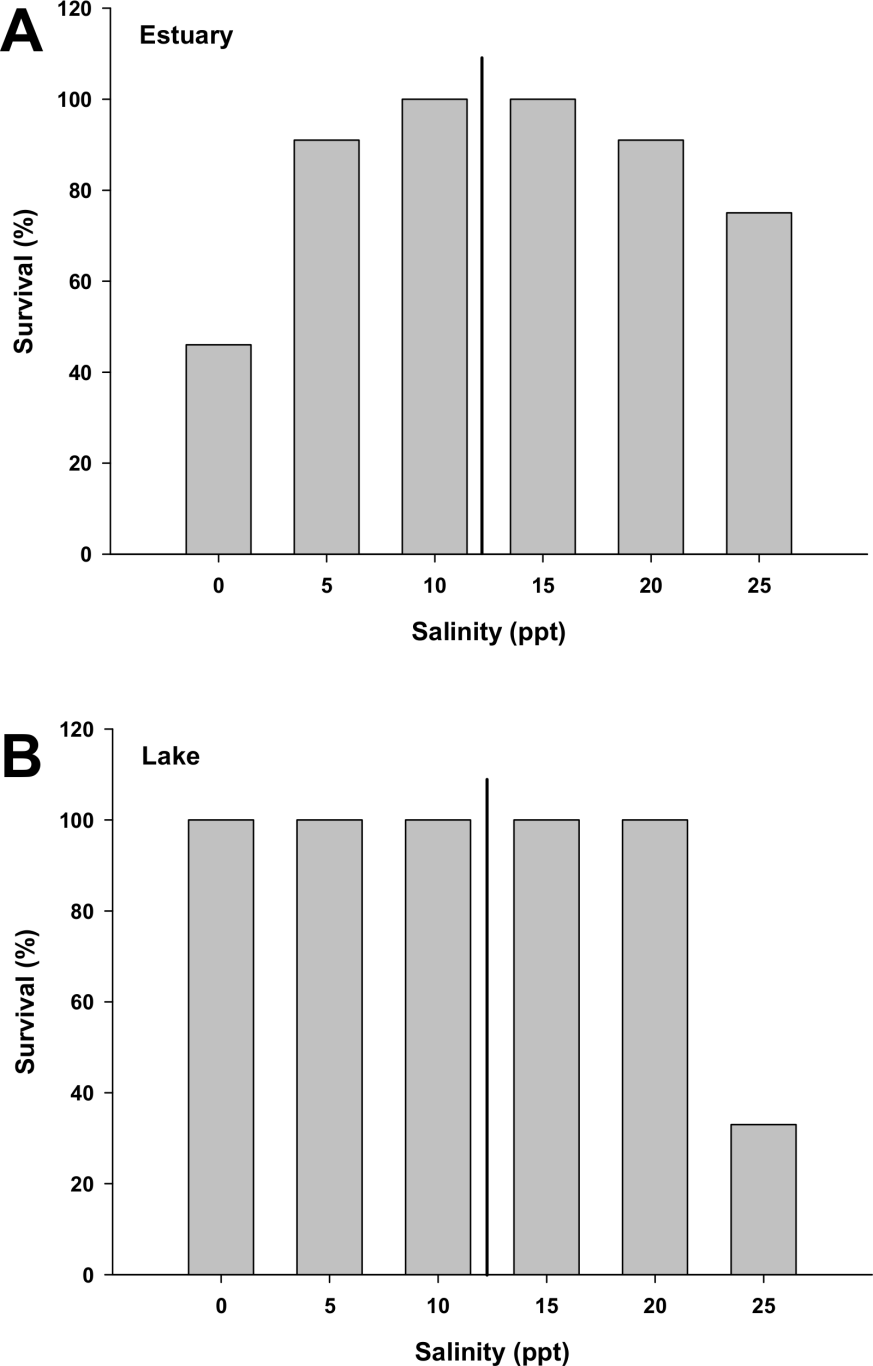
**Survival % of two populations of *G*. *maculatus*:** diadromous (A) and lake-landlocked (B) acclimated to different environmental salinities for 8 days. For each salinity group N = 13 for the estuarine population and N = 9 for the lake population. Vertical black line indicates the iso-osmotic point for this species.

The short-term experiment aimed to test the survival capacity after an abrupt transfer to different environmental salinities. *G*. *maculatus* individuals from a landlocked population (Colico Lake) present a survival rate of 100% after 3 days in a range from 0 to 15 ppt salinity. On the other hand, individuals from a diadromous population (Valdivia river estuary) achieved to acclimate without casualties in a range from 5 to 25 ppt salinity. These results proved to be the first step to the acclimation of the species to a wider range of salinities that goes from freshwater (FW, 0 ppt) to local seawater salinity (SW, 30 ppt). After a gradual acclimation to the final environmental salinities, fish were maintained for another 8 days in them, as it was proved to be enough time for the osmoregulatory branchial cells to reach their new biochemical homeostatic point in this species[24]. Full-strength SW results in 100% mortality for both populations. The survival percentage in the range from 0 to 25 ppt is shown in Fig 4. Briefly, the landlocked population was able to acclimate for 8 days in the range from 0 to 20 ppt without suffering any casualties, while 67% of the population died at 25 ppt. Further enzyme analyses for this population at 25 ppt only include the results of one individual, as only three individuals from this group manage to survive until the start of the experiment, but leave aside from the statistical analysis the results from this group. The estuarine population mortality behavior differs from the previous one as only the individuals acclimated from 10 to 15 ppt managed to survive them all. In this group, the mortality increased as the salinity differs from those optimal salinities (10 to 15 ppt), reaching mortalities of 25% in 25 ppt, and 54% in FW.

The biochemical characterization of the branchial NKA revealed the optimal concentrations for those molecules necessary to fulfill the analysis of this enzyme activity. In this sense, the optimal conditions calculated for *G*. *maculatus* juveniles were 35°C, 7.4 pH, 100 mM Na^+^, 30 mM K^+^, 5 mM Mg^2+^, 0.5 mM ATP and0.5 mM Ouabain. Those concentrations and conditions were used for the analyses of the ATPases activity in all the tissues tested in the present study. Furthermore, gill homogenates were incubated in a range of temperatures from 17 to 47°C, and the results were plotted according to the Arrhenius equation (data not shown). This plot indicated that there is only one relevant isoform for the NKA in the gills of *G*. *maculatus* within the range of temperatures from 23 to 47°C, presenting its maximum of activity at 35°C (which was then considered as the optimal incubating temperature for further analyses of the ATPase enzymes in this species). This branchial enzyme presents an activating energy of 40.9 kJ/mol. Moreover, it looks like there is another NKA isoform that is more active at temperatures lowered than 23°C, but no analyses were done below 17°C, so that its activating energy could not be calculated.

**Fig 5:**
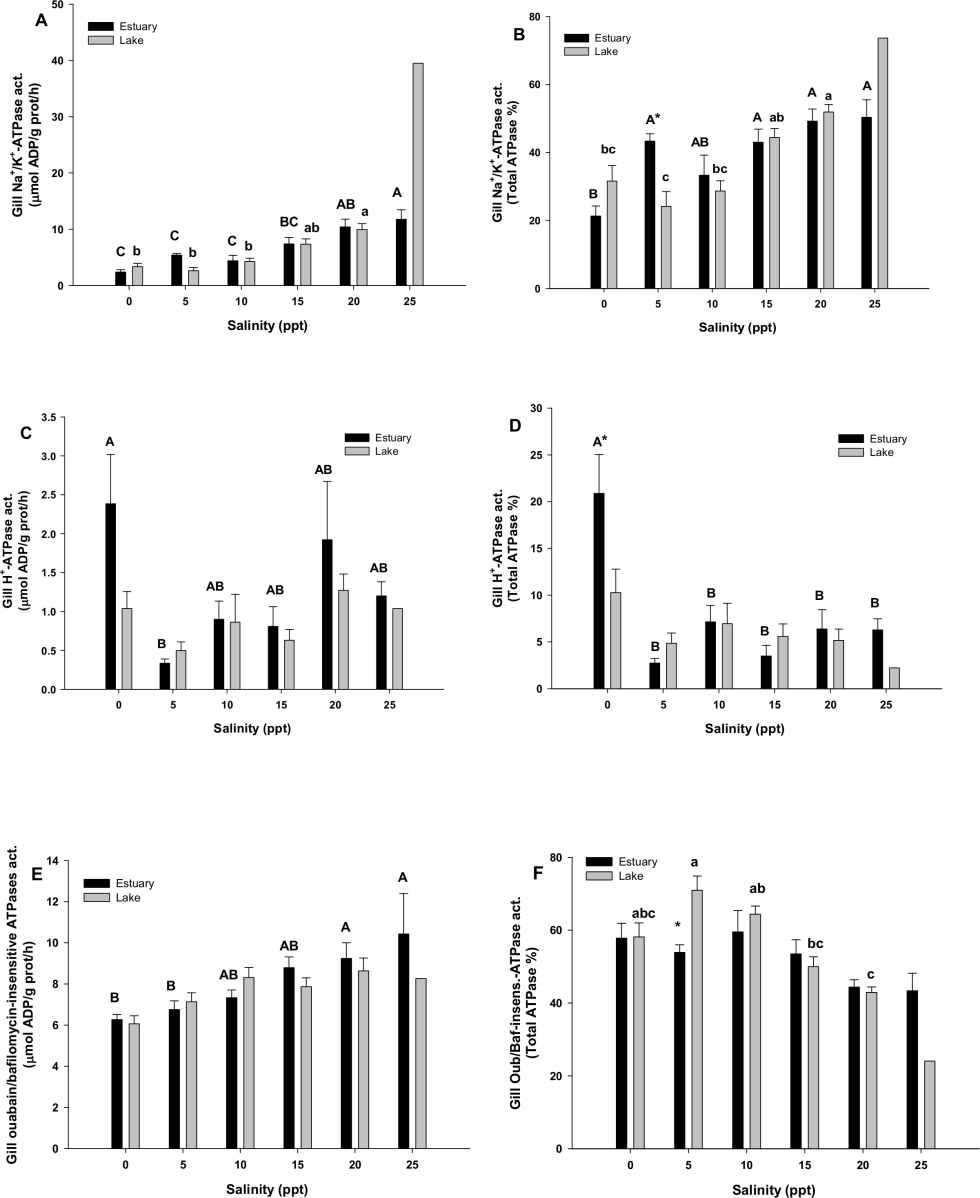
**Branchial Na**^**+**^**/K**^**+**^**-ATPase, H**^**+**^**-ATPase and Ouabain/Bafilomycin-insensitive ATPases activities:** (A, B and C) and % from the total ATPase activity (D, E and F) in *G*. *maculatus* juveniles, from two separated populations (diadromous from an estuary, black bars; and freshwater from a lake, grey bars), acclimated to different environmental salinities for 8 days. Values are mean ± SEM (N = 6, except for the Lake population, N = 1). Different letters indicate significant differences between groups. Major letters refer to the estuary population, while lowercase letters do it for the lake population. Asterisks (*) indicate significant differences between populations at the same environmental salinity (Two way ANCOVA, p<0.05).

Branchial activity of ATPases and their relative % respecting the total amount of ATPases, is shown in Fig 5, with the Na^+^/K^+^-ATPase (NKA) (Fig 5A and 5B), H^+^-ATPase (HA) (Fig 5C and 5D) and the remaining ATPases (Ouabain- and Bafilomycin-insensitive ATPases) activity (Fig 5E and 5F). NKA activity behaves similarly in the gills of the two populations (Fig 5A), presenting its maximum of activity at the highest environmental salinity (25 ppt) and the lowest in the FW groups. This increase is almost linear for the estuarine population from 0 to 25 ppt (r^2^ = 0.924), while in the lacustrine one gill NKA activity remains with constant and low values from 0 to 10 ppt, increasing them from 15 to 20 ppt. Two way analysis comparisons between estuary and lake groups acclimated to the same salinities show that only those submitted to 5 ppt were statistically different (p<0.05), being the diadromous population NKA activity higher than the landlocked one (Fig 5A and 5B). Branchial HA activity (Fig 5C) of the estuary population shows a statistically significant increase at 0 ppt in comparison to the other environmental salinity groups. No differences were shown in the HA activity in the gills of the lake population. Ouabain/Bafilomycin-insensitive ATPases (Fig 5E) of both populations increase linearly with salinity (r^2^ = 0.974 and r^2^ = 0.814 for the estuary and lake populations, respectively).

To better understand the behavior of the branchial ionic pumps, the percentage of activity of every enzyme analyzed was calculated respecting the total ATPases activity at each salinity. Fig 5B represents branchial NKA activity as % of total ATPase activity for each combination of population and salinity. The proportional contribution of NKA is smallest at the lowest salinity levels enzyme, increasing from about 20% to up to 50% of ATPases as salinity increases. Furthermore, NKA enzyme reaches its lowest values in the range from 0 to 10 ppt for the lake population (24–32% of the total ATPases) against more than 45% in the groups acclimated to 15 and 20 ppt salinity.The % of HA (Fig 5D) reveals no major changes respecting to the activity of this enzyme (Fig 5C). More striking results are the % of other ATPases (Fig 5F), as this % does not vary between groups for the diadromous population from the estuary, while increased significantly in the groups maintained from 0 to 10 ppt of the lake population respecting to those at 15 and 20 ppt salinity.

**Fig 6:**
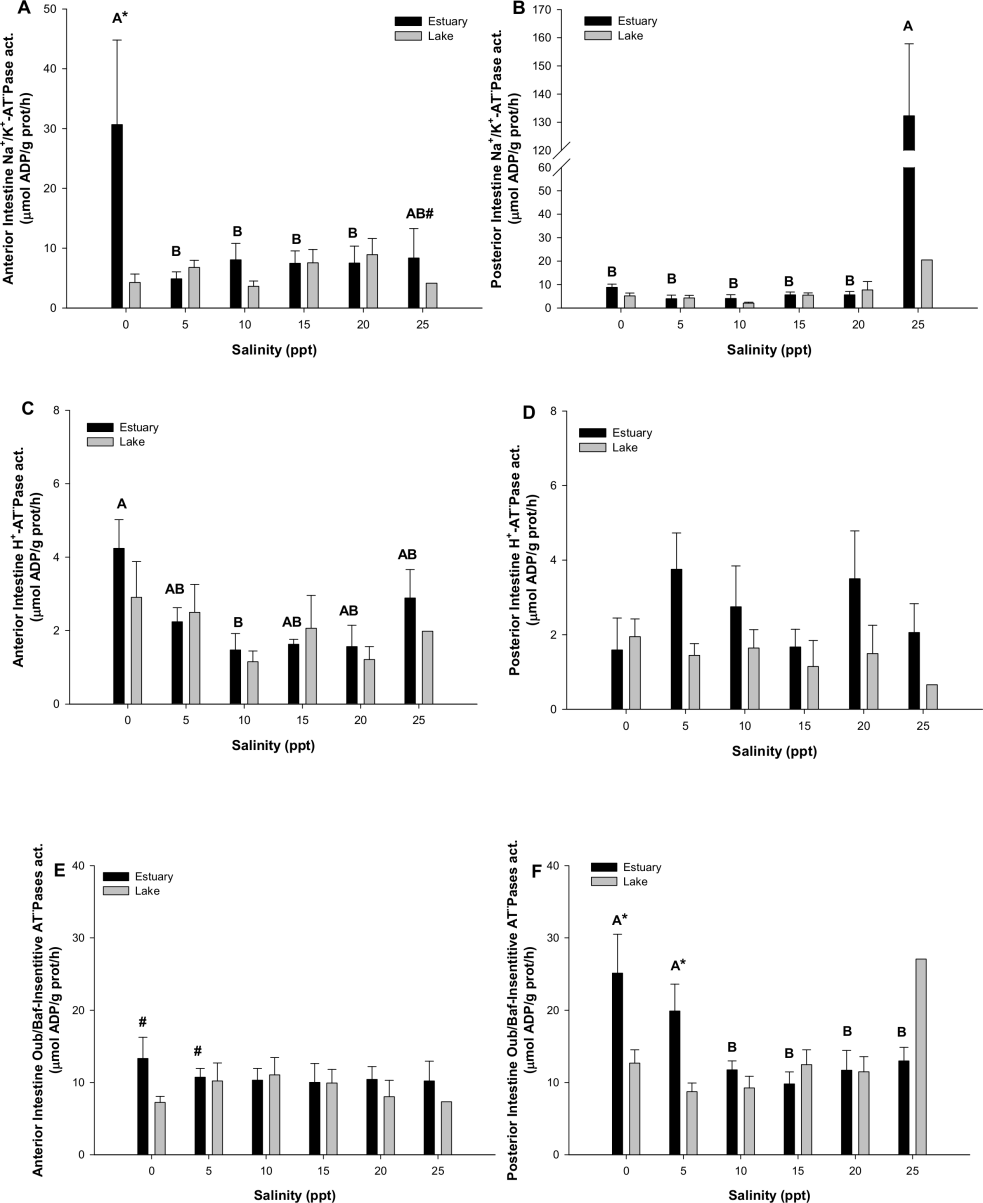
**Anterior intestine Na**^**+**^**/K**^**+**^**-ATPase, H**^**+**^**-ATPase and Ouabain/Bafilomycin-insensitive ATPases activities:** in *G*. *maculatus* juveniles (A, B and C) and posterior intestine Na^+^/K^+^-ATPase, H^+^-ATPase and Ouabain/Bafilomycin-insensitive ATPases activities on (D, E and F), from two separated populations (diadromous from an estuary, black bars; and freshwater from a lake, grey bars), acclimated to different environmental salinities for 8 days. Different letters indicate significant differences within the same intestinal region at different salinities. Major letters refer to the posterior part, while lowercase letters do it for the anterior part. Asterisks (*) indicate significant differences between intestine regions at the same environmental salinity. # indicates significant differences at the same intestinal region and salinity between both populations (Three way ANCOVA, p<0.05). Further details as in legend of Fig 5.

Regarding the intestine ATPases activity, Fig 6 shows the variations of NKA, HA and other ATPases in the anterior part of the intestine in the estuary-lake populations (Fig 6A, 6C and 6E) and posterior part of the intestine in the estuary-lake populations (Fig 6B, 6D and 6F). NKA activity in both parts of the intestine behaves similarly within the range of salinities that goes from 5 to 20 ppt (Fig 6A and 6B), but in the most extreme salinities, it is modified. In this sense, NKA increased significantly (p<0.05) its activity in the anterior part of the intestine in FW respecting to the other salinity groups (from 4.9–8.4 to 30.7μmol ADP/g prot/h), being this group also significantly higher than its counterpart of the lake population. Something similar happens in the posterior part of the intestine, as it increases significantly (p<0.05) its NKA activity in the group acclimated to 25 ppt respecting to the others (from 2.1–7.7 to 132.3 μmol ADP/g prot/h).The activity of the HA behaves similarly (with no statistical differences between groups or intestinal regions) within the range of 10 to 25 ppt of salinity (Fig 6B). Moreover, HA increased its activity in hyposmotic environments, but it does it differently depending on the intestinal region analyzed. In this sense, the anterior part only increased the HA activity at 0 ppt when compared to the other salinity groups (p<0.05), while the posterior region did it at 5 ppt (but no statistical differences, Fig 6C and 6D), did not existing statistical differences between both regions at those salinities. Posterior part of the intestine, in animals acclimated to 5 ppt of the estuary population, revealed a HA activity significantly higher than its counterpart of the lake population, but without statistical differences (Fig 6D) (p<0.05). Other ATPases activity are shown in Fig 6E and 6F, indicating that the anterior part of the intestine does not present differences between groups, unless the group acclimated to 0 ppt presents higher activity values than its counterpart of the lake population. However, the posterior region of the intestine presents a significant increase (p<0.05, from 9.2–13.0 to 21.4μmol ADP/g prot/h) in other ATPases (different than the NKA or the HA) in the salinities of 0 and 5 ppt when compared to any higher salinity. In addition these two salinities (0 and 5 ppt) were statistically different between both intestine portions (p<0.05).

## Discussion

Two populations of *G*. *maculatus* separated for about 10,000 years, one diadromous and the other landlocked in a freshwater (FW) lake in the Chilean Andes, were studied and for the first time the evolutionary drift of the osmoregulatory system in a fish species from the Southern Hemisphere is described. Moreover, phylogeographic analyses permitted to evaluate the timing of the colonization of the Colico Lake, and therefore the onset of this habitat shift.

### Genetic comparisons and demographic inference

During the last decades molecular studies have become very important to further understand and unravel how Quaternary glacial cycles affected the distribution and demography of populations, species, and communities [31, 32]. Evidence of postglacial recolonization in South America has been recorded in freshwater [20, 33] and marine fishes [34], amphibians[35], mammals[36] and marine invertebrates[37, 38]. During glacial maxima species would have become restricted to glacial refugia located outside the influence of glacial ice advances. After this, they expanded their distributions towards previously glaciated areas following the deglaciation processes [32]. Following this, refugial areas are expected to exhibit higher levels of genetic diversity than glaciologically altered or newly founded regions. On the contrary, glaciated areas are expected to show evidence of recent postglacial demographic expansion[39]. In this context, the patterns of genetic diversity and structure recorded in *Galaxias maculatus* match with the expectations of this simple Expansion-Contraction model of Pleistocene biogeography [32]. On the one hand, the Valdivia River population of *G*. *maculatus*, located outside the influence of ice advances, exhibited higher levels of genetic diversity and a very expanded genealogy, which are evidences of an older demographic history. On the other hand, during the Last Glacial Maximum the Colico Lake was covered by ice and melted between 24.6 and 16.8 ky (P. Moreno, personal communication). Glaciologically altered and newly founded areas, as the Colico Lake, exhibit lower levels of genetic diversity, the presence of a dominant haplotype, low divergence among their haplotypes, which are evidence of a younger demographic history due to a recent post-glacial colonization process. In this context, demographic inference analyses suggested that the population expansion of *G*. *maculatus* in the Colico Lake occurred ~ 7,000 years ago, which would also correspond to their shift in habitat and osmoregulatory mechanisms adaption.

### Salinity challenge

The results of the present experiment indicated that the FW population is able to afford a wider range of environmental salinities than the diadromous without suffering any fatalities (0 to 20 ppt for the FW-population *versus* 10 to 15 ppt for the diadromous-population after 8 days of acclimation). Thus, the juveniles from the estuarine population could be considered as stenohaline, as they only survive them all for more than 8 days in a range close to their iso-osmotic point. This iso-osmotic point is, according to previous studies [24, 40], around 10 to 12 ppt of salinity for these species at the life stage of the present study. In this sense, the survival curve presents a Gaussian distribution centered around12 ppt, with higher mortalities as the external salinity increased or decreased. It results intriguing to highlight that the landlocked population only presents mortality above the salinity of 25 ppt. This is apparently the upper limit of survival for this stock, as most individuals died within a few hours at this salinity. This is the first evidence for some osmoregulatory system modifications in about 5 to 10 thousand generations, as this species spawn at the age of one to two years (living up to a maximum of 4 years in certain lakes) [6], with an average calculated rate of 0.5–1 generations/year.

### FW colonization

It is not surprising to find out that the landlocked population of *G*. *maculatus* is able to cope better than the diadromous to the challenge of surviving to a wide range of environmental salinities within just 8 days of acclimation. It was previously stated that the evolution of plasticity could accelerate adaptation during extraordinary environmental changes[5], such as being landlocked in a freshwater lake. However, studies performed in other temperate teleosts, such as the three spine stickleback and the alewife, concluded that landlocked populations of these species presented loss of osmoregulatory plasticity [41–43]. In our case, the osmoregulatory plasticity could arise via selection in less than 10,000 years favoring the more extreme phenotype in the novel environment. Such a process would enable landlocked populations to survive radical habitat shifts.

Previous studies in *G*. *maculatus* have indicated that lacustrine non-migratory populations present a greater genetic structuring than those with access to the sea [4], as our results have confirmed. The genealogy of haplotypes obtained here exposes a recent expansion process, indicating that landlocked individuals in the Andean Colico Lake originally evolved from the same population than those from the Valdivia River Basin. In such dramatic circumstances of being landlocked, the isolated population may evolve into different body morphologies[44], feeding habits [45, 46] or behavioural changes [8].In addition to those, or other variations, the population of the Colico Lake seems to have modified its osmoregulatory strategies.

### Branchial osmoregulatory modifications

The gills, in a process driven by NKA, are the key organ for excretion of ions absorbed through the intestine in SW-acclimated fish, and take up ions from the external media in iso- or hyposmotic environments [17].It has been described that gill genome responses support the hypothesis which regulatory mechanisms are particularly relevant for enabling extreme physiological flexibility [47]. While analyzing the branchial NKA activity, our results are in agreement with those previously described for other species, as this enzyme increased its activity at higher salinities [40, 48], thus suggesting a direct involvement in the excretory processes through the gill epithelium in hyperosmotic environments. Thereby, ionocytes and osmoregulatory cells are restructured according to the environmental salinity, thus modifying their ionic pumps population and ion transport fluxes between FW and SW habitats [10].In this regard, the HA appears to be essential for the ion uptake in freshwater/low salinity environments [48, 49], as our results from the diadromous population confirm. It may be possible that the extra energetic costs derived from this situation triggered *G*. *maculatus* to a process of physical strain that lead to death.

The Colico Lake individuals do not increase their branchial HA activity in FW or any other salinity. However, at salinities below 10 ppt, another population of osmoregulatory enzymes is apparently involved. In particular, when analyzing Ouabain/Bafilomycin-sensitive ATPase enzymes it is revealed that other enzymes rather than the NKA or the HA increase in importance (% of the total ATPase activities) below the isosmotic point (*i*.*e*. 12 ppt) in this population. It would be reasonable to assume a putative role of Ouabain/Bafilomycin-insensitive mechanisms in ion uptake from the external media. As this fact only occurs in the lake individuals, and not in the estuarine population, we hypothesize that there is a noticeable osmoregulatory divergence between both populations when challenged to hyposmotic salinities, thus modifying their hyper-osmoregulatory strategies, at least at branchial level. On the other hand, some authors have established that landlocked *Salmo salar* have lost some of the unique preparatory upregulation of gill ion transporters associated with the development of hypo-osmoregulatory ability in anadromous salmon [50–52]. Since this has been established, it will be of interest to explore the endocrine control of this process, as osmoregulatory responses are further coordinated by hormones [14, 53].

### Intestine

Intestinal osmoregulatory processes, based on the NKA and HA enzymes, evidence a clear anterior to posterior distribution [16, 54].The present study reveals that in *G*. *maculatus* both regions behave similarly in a wide range of environmental salinities. While *G*. *maculatus* from Colico Lake show no relevant differences between the anterior and the posterior regions of the intestine with regards to NKA and HA, the estuarine population presents important differences depending on the external media. Thus, NKA and HA activities in the estuarine population increased considerably in the anterior intestine in FW, responding to a putative need of ion uptake from ingested water [55] or food, an observation that has not obvious physiological role and could not probably be sign of a maladaptive response which is probably linked to the high mortality of this population at this salinity. Previous studies have shown increases of intestinal of ATPase activities with increasing salinities [56]. Individuals from the landlocked population do not present modifications on these ionic pumps at any region of the intestine. It should also be noted that estuarine individuals increased significantly their NKA activity in the posterior region at 25 ppt of salinity. In this sense, the rectum constitutes an additional source of water absorption in hyper-osmotic environments[16, 54], thus maintaining homeostasis during exposure to hypersalinity. Moreover, the posterior region of the intestine in the estuarine population reveals an increase of Ouabain/Bafilomycin-insensitive ATPases activity (rather than the NKA or the HA) in hyposmotic environments, suggesting a role for this fraction in ion movements probably related to water absorption. The lack of differences in ATPases activity analyzed in the lake population in response to salinity challenge is surprising. This observation may indicate that this population is probably unable to initiate drinking and/or intestinal processing once challenged with high salinities. Drinking in freshwater fish is scarce and has not obvious physiological role [57, 58], upon challenge with increased external salinity fish are expected to increase drinking and the subsequent processing of absorbed water to equilibrate osmoregulation. Part of this process relies on the activation and regulation of electrogenic mechanisms *i*.*e*. NKA and HA, which in the case of Colico Lake was not observed. Once again the disparity of intestinal and branchial responses to salinity challenge and their integration in homeostasis in both populations of *G*. *maculatus* points to changes in the endocrine control underlying the differences in ion-transporting mechanisms. Future molecular approaches are essential for a better understanding of this process.

### Implications in other taxa

While the general idea is that the tropical regions are the main place where the biodiversity is generated [59], temperate regions also stimulate the evolutionary adaptation from seawater to freshwater. This was described for the southern hemisphere in members of the family Galaxiidae [60], while in the northern hemisphere, G*asterosteus aculeatus* has evolved in different phenotypic differences [8]. As the latter species has been broadly studied due to its natural widespread distribution, and its biological peculiarities, it should be of interest to also perform an osmoregulatory analysis like that shown in the present study. This fact would throw more light on the osmoregulatory capacities of teleost fish when forced to evolve from seawater to freshwater for a few thousand generations, using a well-known fish model to counteract our results.

## Conclusions

This study shows marked differences in genetic and phylogeographic diversity between a landlocked and a diadromous population of *G*. *maculatus*, suggesting the existence of geographical isolation of the landlocked population for at least 7 ky. Such genetic isolation is reflected in osmoregulatory strategies when both populations are challenged with different environmental salinities. Their mortality rates could be not accounted for by activity of NKA or HA alone, pointing to deeper changes that probably reach the endocrine system and the integration of osmoregulation in both populations.

## Material and Methods

### Ethics statement

The experiments described herein were performed following the standards of the Guide for the Care and Use of Laboratory Animals of the National Commission of Science and Technology (CONICYT, Chile) and the Universidad Austral de Chile, and comply with the 3R procedures. The Ethics protocol was approved by the Committee on the Ethics for Animal Experimentation of Universidad Austral de Chile and the specimens were captured under the Chilean Legislation Technical Memorandum P.INV N° 427/2011, SUBPESCA. We confirm that the field studies did not involve endangered or protected species, solely to state the fact. To minimize suffering, the humane endpoint samplings were performed under anaesthesia with overdoses of 2-phenoxyethanol (0.1% v/v, Sigma P1126). During the survival study, the minimum number of fish were included in the experimental design to make the data meaningful. Animals were monitored after the salinity challenge every two hours during daylight, night time monitoring was eluded to avoid further stress to the fish. The criteria used to assess animal wellbeing were based on visual observation including swimming, interactions between individuals, positive responses to food and prey (brine shrimps twice a day) and changes in skin coloration. Animals showing abnormal symptoms including darkening of the skin, slow movements, school isolation, refusal to feed, disruptions of the breathing cycle (mouth and operculum) and loss of buoyancy and balance control were humanely euthanized (see above).

### Collecting localizations and animal maintenance

Early post-metamorphic juveniles of *Galaxias maculatus* (4.9 ± 0.1 cm and 0.6 ± 0.1 g, mean ± SEM) were collected from two separated populations (diadromous and landlocked) in the south of Chile (February, 2013). The diadromous population was placed in the Valdivia river estuary (39°53´S, 73°24´W, water temperature variations of 16.5 to 21.0°C and daily fluctuations between 5–20 ppt salinity), while the freshwater was landlocked in the Colico lake basin (39°4´60´´S, 72°0´0´´W, water temperature range from18.0 to 22.5xC, 0 ppt salinity and pH 7.5). In the first location seining was used, while electric fishing equipment was employed (EFKO, model FEG 1000, 1 KW, 150–600 V) in the second one. Specimens were immediately placed in aerated 30 L containers and taken to the fish facilities of the Limnological and Marine Sciences Institute of the Universidad Austral de Chile (Valdivia, Chile), where they were allowed to acclimate for 8 days in 70 L tanks (maintaining the same salinity conditions as their capture points). Less than 1% of mortality occurs during this period. Water conditions (pH, temperature, nitrites, nitrates, ammonia and oxygen) were checked and 20% was changed daily. Feeding was carried out *ad libitum* twice a day with freshly hatched brine shrimps (INVE Aquaculture Nutrition, USA). Natural photoperiod was used (month of March in Valdivia, Chile) and temperature ranging from 17°C to 20°C daily.

### Preliminary osmotic shock test

A preliminary study was conducted in order to test the short-term survival to different environmental salinities. Fish from each population were netted and transferred randomly to 10 L (35 x 20 x 15 cm) tanks containing different salinities (0, 5, 10, 15, 20, 25 and 30 ppt) (N = 3 per group). Those salinities were achieved by mixing dechlorinated tapwater from the city of Valdivia (Chile; pH = 7.3, Na^+^ = 0.4 mM, Cl^-^ = 0.2 mM, K^+^ = 0.04 mM and Ca^2+^ = 0.4 mM) and filtered seawater from the coastal laboratory of Calfuco (Valdivia, Chile). Freshwater population survived from 0 to 15 ppt after 3 days without fatal casualties, while the estuarine individuals manage to do it from 5 to 25 ppt without fatalities in the same period.

### Acclimation to different environmental salinities

Gradual transfer was then performed at a rate of 5 ppt every two days, starting from the previously acclimated for 3 days to 15 ppt of the Colico lake individuals, and 5 or 25 ppt for the estuarine fish, reaching the experimental environmental salinities of 0, 5, 10, 15, 20, 25 and 30 ppt (N = 9 or 13 individuals per group for the lake or estuarine populations, respectively) thus establishing the day 0 of the experiment. Individuals were maintained for 8 days in the final salinities. Water quality conditions were maintained as described. Animals were fasted 24 h prior to the sampling.

### Sampling

Fish were quickly netted, anaesthetized with lethal doses of 0.1% (v/v) 2-phenoxyethanol (Sigma P1126) and sampled. During this procedure, weight and length of the animals was recorded, thus allowing the calculation of the condition factor index (K), which was: K (%) = [Weight (g) / Length (cm)^3] * 100. Moreover, the residues condition index was also calculated as follows[61]: body mass was regressed on body size after the data were appropriately transformed (with Ln) to meet the assumptions of regression; then the residual distances of individual points from this regression line served as the estimators of the condition index. Fish were then euthanized by spinal transection. The complete set of gill arches was excised and dried with absorbent paper to remove the blood. As the intestine of this species do not present appreciable differences due to sphincter constrictions after the stomach, it was divided in two equally long (anterior and posterior regions) portions. Those samples were placed in 100 μL of ice-cold sucrose-EDTA-imidazole (SEI) buffer (150 mM sucrose, 10 mM EDTA, 50 mM imidazole, pH 7.3) for ATPase activities analyses.

### Mitochondrial D-loop markers analyses

Individuals tested for physiological analyses were fixed in ethanol (95%), and DNA was extracted using a salting-out methodology previously by Aljanabi and Martinez [62]. For comparison purposes we include in the molecular analyses a similar number of individuals per locality (Colico Lake = 27; Valdivia River = 28). A partial fragment of the mitochondrial D-loop region was amplified using specific primers GAL-F5’–TAA CTC TCA TTA ACT AAA G– 3’ and GAL-R 5’–TGA TAG TAA AGT CAG CAA GCC– 3’ designed from the complete mitochondrial genome of the species (ACN: AP004104) [63]. PCR amplifications were performed in a 25 μL volume containing 2.5 μL 10X Buffer (50 mM KCl, 10 mM Tris-HCl, pH 8.0), 1.0 μL 50 mM MgCl_2_, 200 mM dNTPs, 0.5 μL of each primer (10 pg/μL), 1 U Taq (Invitrogen), 17.5 μL double-distilled water and 5 ng of DNA. Thermal cycling parameters included an initial denaturation step at 94°C for 5 min, followed by 35 cycles at 94°C for 90 sec, 60.7°C for 90 sec and 72°C for 90 sec, and a final 10 min extension at 72°C. Double-stranded amplicons were purified using QIAquick Gel Extraction Kit (QIAGEN) and sequenced in both directions with an Automatic Sequencer ABI3730 x 1 at Macrogen Inc. (Seoul, South Korea). New D-loop haplotypes sequences of *G*. *maculatus* were deposited in GenBank under the Accession Numbers KX133352—KX133406.

### Genetic diversity and population comparisons in *G*. *maculatus*

D-loop sequences were edited using Proseq v. 3.5 [64] and aligned with ClustalW [65]. We performed a DNA saturation analysis in DAMBE [66] to evaluate how saturation of transitions is accumulated in relation to nucleotide divergence in the entire data set. Levels of genetic polymorphism were estimated using standard diversity indices including number of haplotypes (*k*), number of segregating sites (*S*), haplotype diversity (*H*), average number of pairwise differences (*Π*), and nucleotide diversity (*π*) for each locality and for the entire data set with DnaSP v.5.00.07 [67]. We performed statistical neutrality tests (Tajima’s D and Fu’s F_S_) for each locality and for the entire data set to estimate whether sequences deviate from expectations under a neutral model. We determined the levels of genetic differentiation between the analyzed localities using mean pairwise differences (N_ST_) and haplotype frequencies (G_ST_) following previous studies [68] in Arlequin v. 3.5 [69]. The statistical significance of genetic differences was estimated using permutation tests with 10,000 iterations of haplotype identities.

### Demographic inference in *G*. *maculatus*

Genealogical relationships in *G*. *maculatus* were constructed using Maximum Parsimony networks computed in Hapview (http://www.cibiv.at). To estimate the pattern of demographic history in the species, we compared the distribution of pairwise differences between haplotypes (mismatch distribution) of both localities to the expected distribution under the sudden expansion growth model of Rogers and Harpending [70]. This analysis is a popular method since the amount of nucleotide differences between haplotypes depends on the length of time since they diverged. Finally, we reconstructed past population dynamics through time using a Bayesian Skyline Plot method implemented in BEAST v. 1.7 [71]. For comparison purposes, three models (strict clock, uncorrelated lognormal and uncorrelated relaxed clock) were computed for the main areas here analyzed and compared statistically using a Bayesian factor test [72] with TRACER v. 1.5 (http://beast.bio.ed.ac.uk/Tracer). The uncorrelated lognormal model was the best fit for D-loop data in *G*. *maculatus*. We conducted three independent Bayesian MCMC runs using the GTR + I + G model, previously estimated using MrModeltest v. 2.3 (http://www.abc-se/~nylander), and a specific population level mutational rate estimated for *G*. *maculatus* by [63]. For each locality, three independent runs were made for 50 x 10^6^ generations (sampled every 1000 step), discarding a 10% of the trees as burn-in. The convergence of runs was confirmed with Tracer ensuring a minimum of 1000 effective sampling for each statistics (ESS). The results of the multiple runs were combined using LogCombiner [71]. The median and corresponding credibility intervals of the Bayesian skyline plots were depicted with Tracer.

### Enzyme activities

A biochemical characterization of the Na^+^/K^+^-ATPase (NKA) activity in gill homogenates was determined in microplates using a modification of McCormick’s method [73]. Experimental gills and intestine NKA activity was analyzed with the optimal conditions encountered after this characterization. H^+^-ATPase (HA) activity was measured in the same manner as for the NKA using Bafilomycin A1 as a specific inhibitor of the V-type H^+^-ATPase [74], in a final concentration of 100 nM as it inhibits 100% of this enzyme in rainbow trout (*Oncorhynchus mykiss*) [75] and gilthead seabream (*Sparus aurata*) (Ruiz-Jarabo, unpublished results). Remaining ATPases activity was also recorded by subtracting the activities of NKA and HA. Data were expressed as μmol ADP/g prot/h.

### Statistics

Data were checked for normality, independence and homogeneity of variance. A two way analysis of covariance was conducted to gills;the full model included site (estuary-lake) and salinity (0, 5, 10, 15, 20 and 25 ppt) as fixed effects, as well as all possible interactions between the terms. Intestine was tested by three way analysis of covariance; the full model included site (estuary-lake), salinity (0, 5, 10, 15, 20, 25 ppt) and portion of intestine (anterior and posterior) as fixed effects as well as all possible interactions between the terms. All models included length as a covariate and tank as a random effect. Statistical significance was accepted at p<0.05. All data are presented as mean±standard error (S.E.). This model was chosen from Velotta et al. (2015) [75].Differences in length, weight and Fulton´s condition factor (K) of two different populations (estuary and lake) were analyzed by the Student´s *t*-test.
